# Hypermethylation of *LATS2* Promoter and Its Prognostic Value in *IDH*-Mutated Low-Grade Gliomas

**DOI:** 10.3389/fcell.2020.586581

**Published:** 2020-10-22

**Authors:** Yuan Gu, Yu Wang, Yebin Wang, Jiaqian Luo, Xin Wang, Mingyue Ma, Wei Hua, Ying Liu, Fa-Xing Yu

**Affiliations:** ^1^Institute of Pediatrics, Children’s Hospital of Fudan University and the Shanghai Key Laboratory of Medical Epigenetics, The International Co-laboratory of Medical Epigenetics and Metabolism, Ministry of Science and Technology, Institutes of Biomedical Sciences, Shanghai Medical College, Fudan University, Shanghai, China; ^2^Department of Neurosurgery, Huashan Hospital, Fudan University, Shanghai, China; ^3^Department of Pathology, School of Basic Medical Sciences, Fudan University, Shanghai, China

**Keywords:** low-grade glioma, Hippo pathway, *Lats2*, YAP, IDH1/2, isocitrate dehydrogenase

## Abstract

Mutations in the enzyme isocitrate dehydrogenase 1/2 (*IDH1/2*) are the most common somatic mutations in low-grade glioma (LGG). The Hippo signaling pathway is known to play a key role in organ size control, and its dysregulation is involved in the development of diverse cancers. Large tumor suppressor 1/2 (LATS1/2) are core Hippo pathway components that phosphorylate and inactivate Yes-associated protein (YAP), a transcriptional co-activator that regulates expression of genes involved in tumorigenesis. A recent report from The Cancer Genome Atlas (TCGA) has highlighted a frequent hypermethylation of *LATS2* in *IDH*-mutant LGG. However, it is unclear if *LATS2* hypermethylation is associated with YAP activation and prognosis of LGG patients. Here, we performed a network analysis of the status of the Hippo pathway in *IDH*-mutant LGG samples and determined its association with cancer prognosis. Combining TCGA data with our biochemical assays, we found hypermethylation of *LATS2* promoter in IDH-mutant LGG. *LATS2* hypermethylation, however, did not translate into YAP activation but highly correlated with *IDH* mutation. *LATS2* hypermethylation may thus serve as an alternative for *IDH* mutation in diagnosis and a favorable prognostic factor for LGG patients.

## Introduction

Mutations in isocitrate dehydrogenase 1 and 2 (*IDH1/2*), mainly Arg132 for *IDH1* and Arg140 and Arg172 for *IDH2*, occur in over 80% of low-grade glioma (LGG) (Parsons et al., 2008; Watanabe et al., 2009; Yang et al., 2012; Cancer Genome Atlas Research Network et al., 2015; Suzuki et al., 2015). While wild type IDH1/2 converts isocitrate to α-ketoglutarate (αKG), gain-of-function mutations of *IDH1/2* lead to the production and accumulation of oncometabolite R-2-hydroxyglutarate (R-2HG) (Zhao et al., 2009; Ward et al., 2010). R-2HG drives tumorigenesis by inhibiting αKG-dependent enzymes involved in epigenetic modifications, response to hypoxia, and other biological processes (Zhao et al., 2009; Figueroa et al., 2010; Chowdhury et al., 2011; Xu et al., 2011; Lu et al., 2012; Turcan et al., 2012).

The Hippo pathway consists of a kinase cascade and plays crucial roles in tissue homeostasis and tumorigenesis (Harvey et al., 2013; Yu and Guan, 2013; Moroishi et al., 2015; Yu et al., 2015; Patel et al., 2017; Luo and Yu, 2019). Hippo pathway activation results in the phosphorylation of core Ste20-like kinases MST1 and MST2 (MST1/2), which phosphorylate and activate large tumor suppressor 1/2 (LATS1/2) kinases. LATS1/2, in turn, phosphorylate and inactivate Yes-associated protein (YAP) and WW domain-containing transcription regulator protein 1(WWTR1, also known as TAZ), which function as transcription co-activators and serve as Hippo pathway downstream effectors by regulating expression of genes involved in cell proliferation, death, and differentiation. MST1/2 and LATS1/2 activity is further regulated by diverse regulators and upstream signals. Dysregulation of Hippo pathway has been associated with various cancers (Yimlamai et al., 2015; Yu et al., 2015; Zanconato et al., 2016; Gregorieff and Wrana, 2017; Zhang and Zhou, 2019). *LATS2* deficiency, for instance, has been studied in several cancers including glioma (Kawahara et al., 2008; Guo et al., 2017, 2019; Ye et al., 2017; Jin et al., 2018; Pan et al., 2018; Shi et al., 2018, 2019; He et al., 2019; Hsu et al., 2019).

A recent report from The Cancer Genome Atlas (TCGA) Research Network revealed that the promoter of *LATS2* is hypermethylated in almost all *IDH*-mutated LGG clinical samples but not in *IDH*-wild type samples (Sanchez-Vega et al., 2018). *LATS2* promoter hypermethylation in *IDH*-mutated LGG samples is expected to downregulate *LATS2* expression and subsequently activate YAP/TAZ and expression of downstream target genes. However, this hypothesis has not been systematically analyzed and experimentally tested. Here, combining TCGA data with our biochemical assays, we performed a network analysis of the status of the Hippo pathway in *IDH*-mutant LGG samples and determined its association with cancer prognosis.

## Results

### Promoter Hypermethylation and Low Expression of *LATS2* in *IDH*-Mutant LGG

**FIGURE 1:**
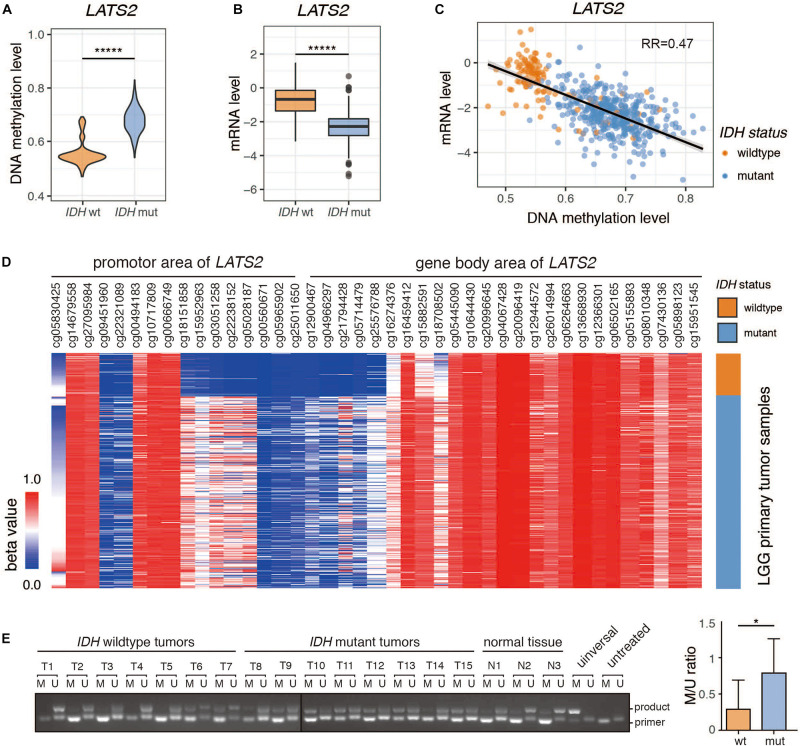
Promoter hypermethylation and low expression of *LATS2* in *IDH*-mutant LGG. **(A)** *LATS2* methylation level is increased in *IDH*-mutant LGG. **(B)** *LATS2* mRNA level is decreased in *IDH*-mutant LGG. **(C)** Correlation between *LATS2* methylation level *and LATS2* mRNA level. RR indicates R squared value of linear regression. **(D)** Methylation level of different CpG islands in *LATS2* promoter and gene body area. **(E)** Methylation-specific PCR of LGG samples. M: methylation-specific primer; U: unmethylation-specific primers; universal: universally methylated genomic DNA control; untreated: untreated U87 cell genomic DNA; product: ∼130 bp PCR products; primer: primer dimers. Quantitative result on the right. M/U ratio was calculated by comparing the bands from methylation-specific and unmethylation-specific primers of each sample. Mean and standard error were presented (*p < 0.05, *****p < 0.000005, *t* test).

We examined *LATS2* methylation level and mRNA expression in LGG dataset from TCGA, and found that *LATS2* promoter was hypermethylated and *LATS2* mRNA was repressed in *IDH*-mutant LGG compared to *IDH*-wild type LGG (Figures 1A,B). Moreover, *LATS2* mRNA levels negatively correlated with methylation levels (Figure 1C). The differences in *LATS2* gene methylation were mainly located within the promoter region instead of gene body (Figure 1D). We also collected LGG specimens with or without *IDH*1/2 mutations, and measured *LATS2* promoter methylation using a methylation-specific PCR assay (Herman et al., 1996; Oh et al., 2015). Consistent with TCGA data, *LATS2* promoter methylation was significantly higher in *IDH*-mutant LGG (Figure 1E). It is worth noting that while *LATS2* promoter hypermethylation had been reported in another cancer with frequent *IDH* mutations, namely *IDH*-mutant acute myeloid leukemia (AML), it did not downregulate *LATS2* expression as it did in LGG (Supplementary Figure 1A), suggesting a different mechanism or role. Meanwhile, while *LATS1* was also hypermethylated, it was not downregulated as *LATS2* (Supplementary Figure 2A). Overall, our results indicate that *LATS2* is hypermethylated and repressed in *IDH*-mutant LGG.

### Hippo Pathway Target Genes Are Not Activated by *LATS2* Deficiency in *IDH*-Mutant LGG

**FIGURE 2:**
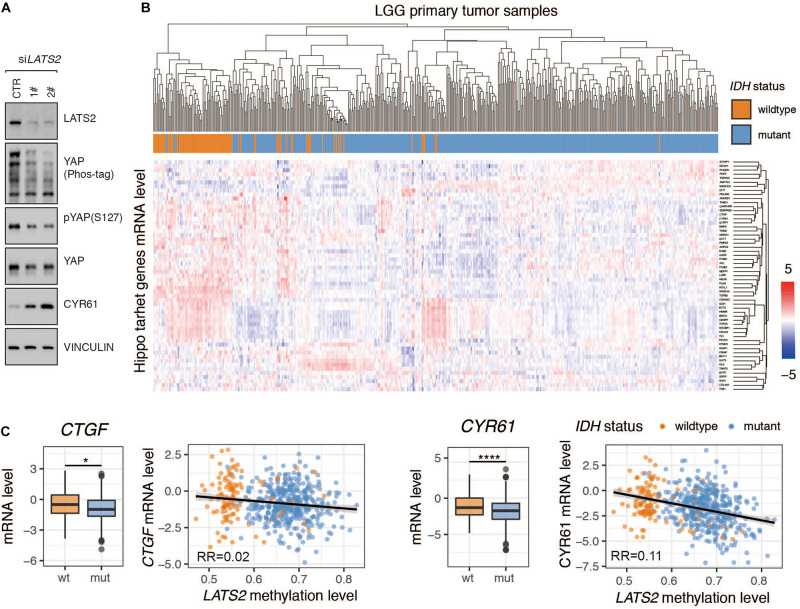
Hippo pathway target genes are not activated by *LATS2* deficiency in IDH-mutant LGG. **(A)** YAP is activated in HEK293A cells with *LATS2* knockdown. **(B)** cluster analysis of Hippo target gene expression in *IDH-*wildtype and mutant LGG. The Normalized RESM value was scaled across each gene to yield standard score (Z-score) **(C)** mRNA levels of Hippo target genes *CTGF* and *CYR61* were decreased in *IDH*-mutant LGG. Correlation between *CTGF*/*CYR61* mRNA level and *LATS2* methylation level is shown. Mean and standard error were presented (*p < 0.05, ****p < 0.00005, *t* test).

Given that LATS2 is a direct upstream regulator of YAP/TAZ, we examined the effects of *LATS2* knockdown on YAP activity. Using two independent siRNAs to target LATS2 in HEK293 cells, we observed that LATS2 knockdown significantly reduced YAP phosphorylation and increased target gene *CYR61* expression (Figure 2A). The same result was also observed in glioma cell lines (Guo et al., 2019; Shi et al., 2019). Hence, silencing LATS2 expression in HEK293 cells led to YAP activation.

Subsequently, we analyzed if Hippo pathway target genes were activated following *LATS2* downregulation in *IDH*-mutant LGG. Surprisingly, the association between Hippo pathway target gene expression with IDH mutation was weak (Figure 2B). For instance, the mRNA levels of *CTGF* and *CYR61* were reduced in *IDH*-mutant LGG samples, and correlation analyses showed a nearly negative correlation between Hippo pathway target gene expression and *LATS2* methylation (Figure 2C). Hence, it appeared that at least in *IDH*-mutant LGG, LATS2 downregulation did not translate to YAP activation and YAP-dependent gene expression.

### Hippo Pathway Target Genes Are Universally Hypermethylated in IDH-Mutant LGG

**FIGURE 3:**
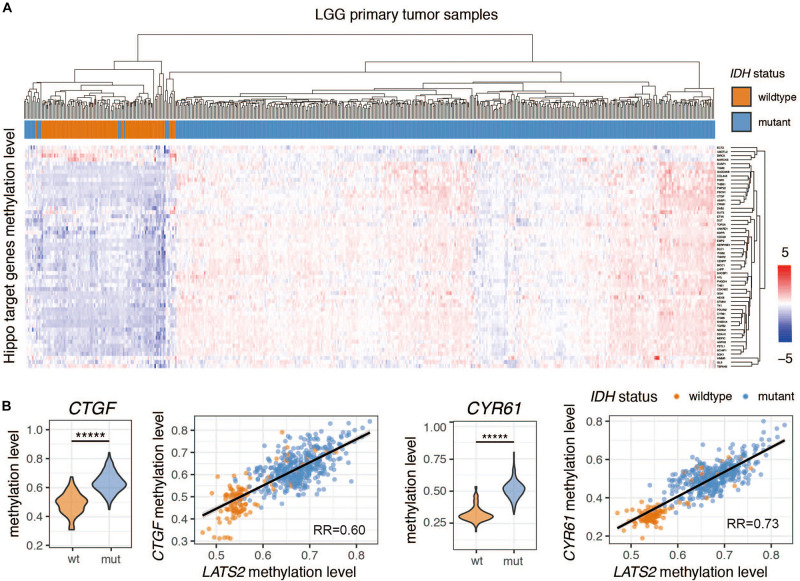
Hippo pathway target genes are universally hypermethylated in *IDH*-mutant LGG. **(A)** cluster analysis of Hippo target gene methylation levels in *IDH-*wildtype and mutant LGG. The Normalized beta value is scaled across each gene to yield standard score (Z-score) **(B)** Methylation levels of Hippo target genes *CTGF* and *CYR61* are significantly increased in *IDH*-mutant LGG. Correlation between *CTGF*/*CYR61* methylation level and *LATS2* methylation level is shown. Compared by *t* test (*****p < 0.000005, *t* test).

The high methylation levels of *CTGF* and *CYR61* in *IDH*-mutant LGG suggested that Hippo pathway target genes were also regulated by methylation (Figure 3B). Indeed, a cluster analysis showed that most Hippo pathway target genes were hypermethylated in *IDH*-mutant LGG samples (Figure 3A). The methylation of these genes was comparable to that of *LATS2*, as indicated by a tight correlation between methylation levels of *LATS2* and those of *CTGF* or *CYR61* (Figure 3B). Thus, the nearly universal hypermethylation of Hippo pathway target genes may explain the ineffectiveness of *LATS2* hypermethylation in *IDH*-mutant LGG to affect YAP activation and target gene expression.

### Low Expression of YAP/TAZ in LGG

**FIGURE 4:**
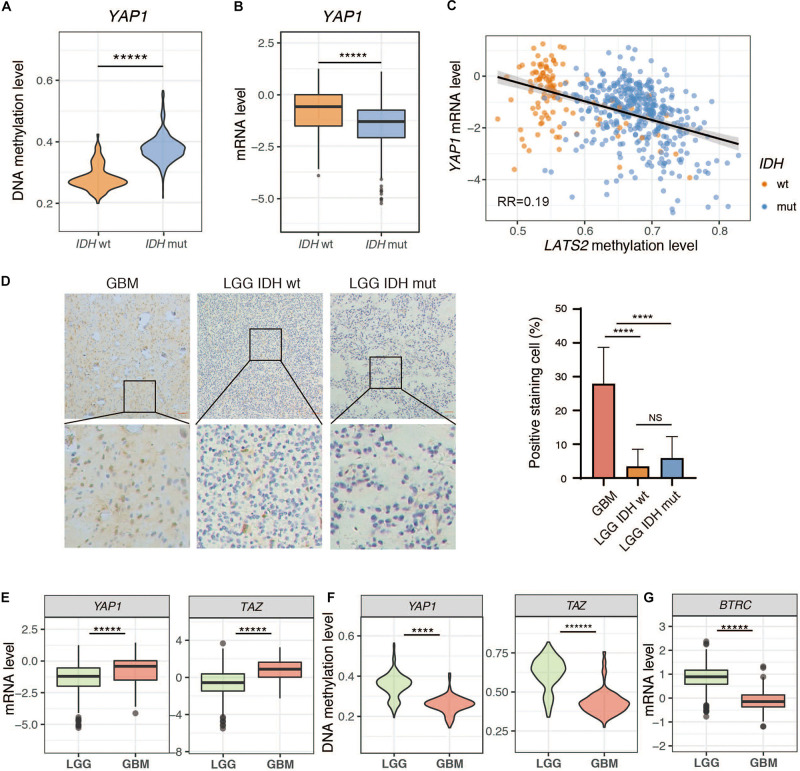
Low expression of *YAP* in LGG. **(A)** *YAP* methylation is elevated in *IDH*-mutant LGG. **(B)** *YAP* mRNA level is reduced in *IDH*-mutant LGG. **(C)** Correlation between *YAP* mRNA level and *LATS2* methylation level. **(D)** IHC result of *IDH-*wildtype or mutant LGG samples and GBM samples. Left: Representative image; Right: Quantitative result. **(E)** *YAP* and *TAZ* mRNA levels in LGG and GBM. **(F)** *YAP* and *TAZ* methylation levels in LGG and GBM. **(G)** *BTRC* mRNA level in LGG and GBM. Mean and standard error were presented (****p < 0.00005, *****p < 0.000005, *t* test).

We next analyzed the expression of YAP and TAZ in LGG. Interestingly, both *YAP* and *TAZ* were hypermethylated, and were expressed at lower levels in *IDH*-mutant LGG samples compared to *IDH*-wild type LGG samples (Figures 4A–C and Supplementary Figure 3). We then assessed YAP expression by immunohistochemistry (IHC) in LGG tumor specimens. Our IHC results indicated, however, that YAP expression was either absent or extremely weak in all LGG samples, regardless of *IDH* status. In contrast, YAP was highly expressed in glioblastoma (GBM), another common brain tumor (Figure 4D). This could be due to overall higher methylation and lower expression of *YAP* in LGG compared to GBM (Figures 4E,F), although it could not explain why YAP protein expression showed no significant difference between *IDH*-wild type and *IDH*-mutant LGG samples. Hence, it is possible that a posttranslational mechanism may account for low YAP protein levels in LGG. Intriguingly, we found that BTRC, an E3 ligase responsible for YAP degradation (Zhao et al., 2010), was dramatically upregulated in LGG compared to GBM (Figure 4G). On the other hand, several reported deubiquitinases for YAP (Li et al., 2018; Sun et al., 2019; Zhang et al., 2019; Pan et al., 2020; Zhu et al., 2020) were also upregulated (Supplementary Figure 4). Thus, further work is needed to dissect the mechanism(s) for the loss of YAP protein expression in LGG.

### Dysregulated Expression of Multiple Hippo Pathway Genes in *IDH*-Mutant LGG

Since *LATS2*, *YAP*, and several Hippo pathway target genes were highly methylated in IDH-mutant LGG, we analyzed methylation and gene expression of known Hippo pathway components in LGG. We found that many of them are dysregulated in IDH-mutant LGG (Supplementary Figures 5–8, summarized in Supplementary Figure 9).

**FIGURE 5:**
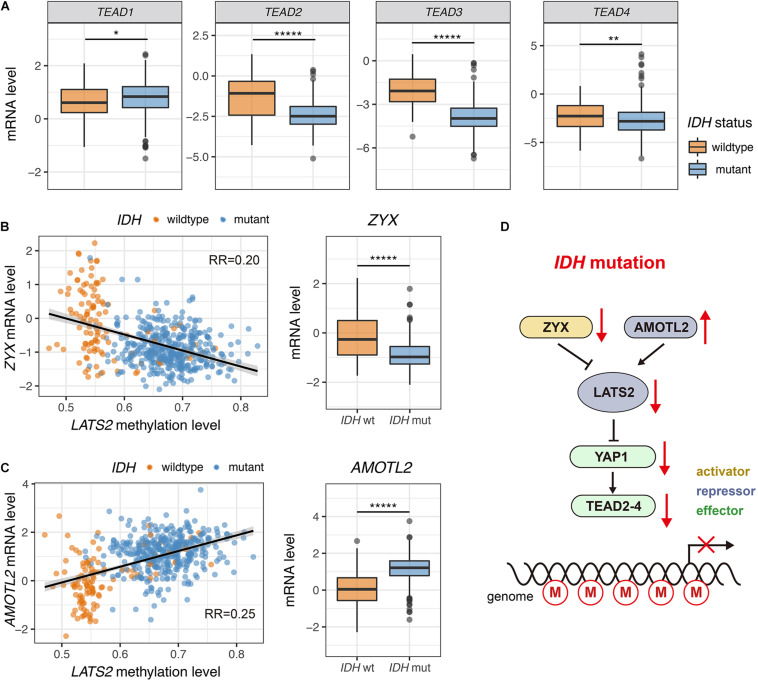
Dysregulated expression of multiple Hippo pathway genes in *IDH*-mutant LGG. **(A)** TEAD2-4 are significantly downregulated in *IDH*-mutant LGG while TEAD1 shows a mild upregulation. **(B)** Correlation between *ZYX* mRNA level and *LATS2* methylation level (left). *ZYX* is significantly downregulated in *IDH*-mutant LGG (right). **(C)** Correlation between *AMOTL2* mRNA level and *LATS2* methylation level (left). *AMOTL2* is significantly upregulated in *IDH*-mutant LGG (right). **(D)** A proposed model of ineffective LATS2 hypermethylation. Mean and standard error were presented (*p < 0.05, ** < 0.005, *****p < 0.000005, *t* test).

TEA-domain family proteins (TEAD1-4) are the major transcription factors that mediate functions of YAP/TAZ (Ota and Sasaki, 2008; Chen et al., 2010; Lamar et al., 2012; Lin et al., 2017; Holden and Cunningham, 2018). Notably, *TEAD2-4* were significantly downregulated in IDH-mutant LGG, while *TEAD1* showed a mild upregulation (Figure 5A). Low expression of TEAD genes was correlated with *LATS2* hypermethylation (Supplementary Figure 5).

Further, we observed that the expression of known upstream regulators of LATS1/2 were modulated in IDH-mutant LGG samples. For instance, the mRNA levels of Zyxin (ZYX), an inhibitor of LATS2 (Ma et al., 2016), were low in IDH-mutant LGG samples (Figure 5B). On the other hand, the expression of angiomotin like 2 (AMOTL2), an activator of LATS2 (Mana-Capelli and McCollum, 2018), was elevated in IDH-mutant LGG samples (Figure 5C). These changes may also play a role in restricting YAP/TAZ activity in IDH-mutant LGG by inducing activity of residual LATS1/2 (Figure 5D).

### *LATS2* Hypermethylation Is a Favorable Prognostic Factor in Overall LGG but Not in *IDH*-Wild-Type or Mutant Subgroups

**FIGURE 6:**
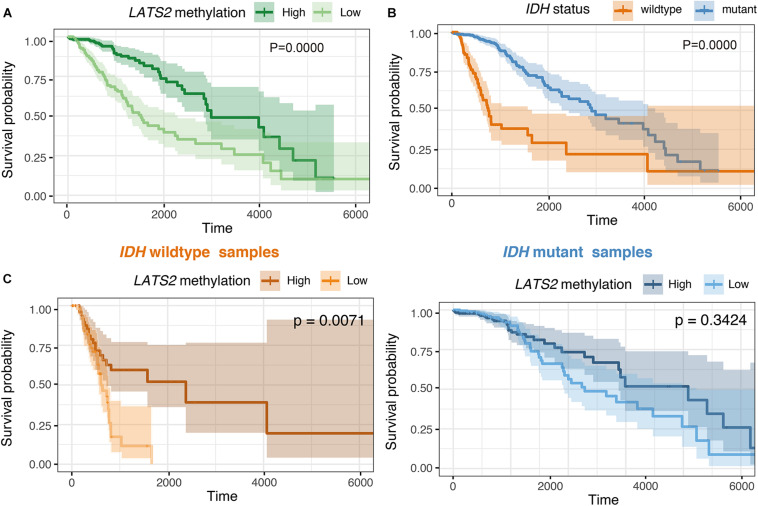
*LATS2* hypermethylation is a favorable prognostic factor in overall LGG. **(A)** High *LATS2* methylation is a favorable prognostic factor in overall LGG. *P* value as indicated. **(B)** *IDH* mutation is a favorable prognostic factor of overall LGG; **(C)** High *LATS2* methylation level is a favorable prognostic factor in *IDH*-wildtype LGG but not in *IDH-*mutant LGG.

Our results thus far indicated that the hypermethylation of *LATS2* in *IDH*-mutant LGG failed to activate YAP/TAZ activity. Next, we interrogated whether hypermethylation of *LATS2* could serve as a biomarker with clinical significance. In analyzing survival data of LGG patients, we found that *LATS2* hypermethylation is a strong favorable prognostic factor in LGG (Figure 6A). However, when we performed analysis separately in *IDH*-mutant patients, *LATS2* hypermethylation showed no prognostic significance in *IDH*-mutant subgroups (Figure 6C). In comparing the clinical features between high and low *LATS2* methylation groups, we uncovered several characteristics that varied between these two groups including *IDH1/2* status (Supplementary Table 2). As *IDH* mutation was a favorable prognostic factor of LGG (Vuong et al., 2019; Figure 6B), the prognostic significance of *LATS2* hypermethylation was likely due to its enrichment in *IDH*-mutant samples.

To further explore the prognostic value of *LATS2* methylation, we did Cox proportional hazard analysis of *LATS2* methylation (Table 1). We found *LATS2* methylation is a prognostic factor in overall LGG after adjusted for a series of covariates, but lost its prognostic value in either *IDH*-wildtype or mutant LGG subgroups, indicating its prognostic significance comes from correlation with *IDH* mutation instead of direct impact on Hippo pathway effectors.

**TABLE 1:** Cox proportional hazard model of *LAST2* methylation and expression in LGG patients.

	Overall		*IDH* wildtype		*IDH* mutant	
Variable	Univariable	Adjusted*	Univariable	Adjusted*	Univariable	Adjusted*
	HR (95%CI)	HR (95%CI)	HR (95%CI)	HR (95%CI)	HR (95%CI)	HR (95%CI)
*LATS2* methylation	**1.64e-05**	**3.63e-04**	**1.32e-05**	2.72e-02	0.37	6.69e-03
	**(1.06e-6, 2.53e-4)**	**(2.28e-5, 5.78e-3)**	**(1.09e-8, 1.60e-2)**	(2.39e-6, 309.82)	(1.99e-3, 68.60)	(2.64e-5, 1.70)
*IDH* status wildtype	**4.80**	**3.36**				
	**(3.34, 6.90)**	**(2.18, 5.18)**				

*Adjusted for age, gender, neoplasm histologic grade, histological type, and tumor location. HR with *p* value < 0.05 is marked by bold formatting, CI denotes confidence interval.

### LATS2 Hypermethylation Predicts IDH Mutation in LGG

Lastly, we determined whether *LATS2* methylation level may work as a biomarker of *IDH* status. Using CpG island cg03051258 in the promoter area of *LATS2* as an example, we applied beta value 0.1 as a threshold to identify *LATS2* hyper- and hypo-methylated samples, and IDH-mutant LGG was successfully enriched in the *LATS2* hypermethylated group. Using this approach, we could predict *IDH* mutation in LGG at the sensitivity of 0.95 and specificity of 0.97 (Table 2). Together, these data support that *LATS2* hypermethylation is a faithful biomarker of *IDH* mutations.

**TABLE 2:** Methylation level of cg03051258 to predict *IDH* status.

cg03051258	*IDH* mutant	*IDH* wildtype	Total
beta value > 0.1	393	3	396
beta value < 0.1	19	88	107
Total	412	91	503

## Discussion

The Hippo pathway is known to play critical roles in cancer development, making this signaling network an area of high clinical interest. The TCGA Network project revealed that *LATS2* is commonly hypermethylated in *IDH*-mutant low-grade gliomas, prompting us to explore its role in LGG. Several groups have previously explored a role of LATS2 in gliomas (Guo et al., 2019; Shi et al., 2019). However, a systematic analysis to evaluate its effect on Hippo pathway in *IDH*-mutant LGG had not been carried out.

Our study found that *LATS2* promoter was hypermethylated while *LATS2* mRNA was repressed in *IDH*-mutant LGG samples. Unexpectedly, *LATS2* repression failed to activate Hippo pathway target genes, as most of these genes were also hypermethylated in *IDH*-mutant LGG samples. The universal epigenetic changes caused by *IDH1/2* mutation could be a key point to understand this phenomenon. Oncometabolite *R-2HG* produced by mutated IDH1/2 inhibits the activity of αKG-dependent enzymes including DNA and histone demethylases (Yamane et al., 2006; Chowdhury et al., 2011; Ito et al., 2011; Xu et al., 2011; Turcan et al., 2012; Kohli and Zhang, 2013). In this way, *IDH1/2* mutation may cause genome-wide alterations in DNA methylation, including *LATS2*, Hippo pathway target genes, and additional Hippo pathway component genes.

It is interesting that YAP expression is high in GBM but is extremely low in all LGG regardless of *IDH* status. This could be a reflection of differentiation status and aggressiveness of tumors, because YAP is frequently activated in less differentiated and malignant cancers (Xu et al., 2009; Fullenkamp et al., 2016; Zanconato et al., 2016). Compared to GBM, LGG is usually well-differentiated and less malignant, and YAP may remain less active in LGG. Moreover, YAP is important in maintaining stemness of progenitor cells (Lian et al., 2010; Beyer et al., 2013; Li et al., 2013). Hence, the difference in YAP activity between GBM and LGG might be inherited from the status of respective cancer progenitor cells.

Along with low YAP expression, additional mechanisms may contribute to the lack of YAP activation in *IDH*-mutant LGG. For instance, the dysregulated expression of *ZYX* and *AMOTL2* may inhibit LATS1/2 activity, while reduction of *TEAD2-4* expression may limit the transcriptional output of YAP.

Although *LATS2* hypermethylation was unable to activate YAP in *IDH*-mutant LGG, it displayed a strong correlation with *IDH1/2* mutation and could serve as a favorable prognostic factor for LGG patients. In addition, *LATS2* hypermethylation was a faithful biomarker of *IDH* mutations, and could potentially be used as an alternative for IDH mutation in diagnosis.

In conclusion, our study found *LATS2* promoter hypermethylation in *IDH*-mutant LGG samples which, surprisingly, did not translate into YAP activation, raising the role and involvement, if at all, of the Hippo pathway in the development of LGG. Meanwhile, *LATS2* hypermethylation showed a strong correlation with *IDH* mutation. Hence, *LATS2* hypermethylation can serve as an alternative for IDH mutation in diagnosis and a favorable prognostic factor for LGG patients.

## Materials and Methods

### Data Collection and Processing

TCGA-LGG, TCGA-GBM, TCGA-AML RNA sequence level 3 normalized data, DNA Methylation Level 3 data, clinical data and somatic mutation data were downloaded from GDC Data Portal using package TCGAbiolinks in R (version 3.6.2) environment for further analysis (Colaprico et al., 2016). *IDH*-mutant samples were composed of samples with IDH1 Arg132 or IDH2 Arg140 and 172 mutations. The level 3 expression data were normalized RMSE value. For each gene, we zero-centered expression data by calculating standard score (Z-score) between each individual sample. Comparison of gene expression between different tumor types were based on pan-cancer normalized expression data from UCSC Xena team (Goldman et al., 2020). Pre-process steps to yield level 3 methylation data (*β*-value) included background correction, dye-bias normalization. *β*-values ranged from zero to one, with zero indicating no methylation detected.

### Statistical Analysis

The expression and methylation of Hippo-related genes were compared by *t* test or Mann-Whitney U test. The correlation between expression and methylation status was evaluated by fitting linear models. Survival data were analyzed by Kaplan-Meier analysis and Cox proportional hazard analysis. The Hippo pathway target genes (Supplementary Table 1). were determined according to RNAseq results of our Hippo element knock-out cell lines (data not shown). Hierarchical Clustering of each sample was done by calculating Euclidean distance matrix followed Pearson correlation analysis based on Hippo target features. All the analysis and image drawing (R package ggplot2) was done by R (version 3.6.2).

### Patients Samples

This study enrolled patients with GBM (*n* = 8) and LGG (*n* = 12, including 5 *IDH* wildtype and 7 *IDH* mutant). Glioma frozen tissues and paraffin slides were obtained from Huashan Hospital, Fudan University, Shanghai, China. This study was approved by the Ethics Committee of the Huashan Hospital of Fudan University and informed consents were obtained from all participants. Specimens used for Methylation-specific PCR were taken at the time of surgical resection, snap−frozen in liquid nitrogen and stored at −80°C until use. Formalin-fixed paraffin-embedded (FFPE) tissues were used for immunohistochemical (IHC) staining.

### Cell Culture and Gene Knock-Down by siRNA

HEK293A cells were cultured in DMEM (Corning) containing 5% FBS (Gibco) and 50 μg/mL penicillin/streptomycin (P/S). All cells were incubated at 37°C under 5% CO2. siRNAs were purchased from GenePharma and transfected into cells using Lipofectamine RNAiMAX reagent (Invitrogen) according to the manufacturer’s protocol. The following siRNAs were used: si*LATS2* 1#: UACCAUAAAUACAAUCUUCTT (5′-3′), si*LATS2* 2#: CCGCAAAGGGTACACTCAATT (5′-3′). HEK293A cells were seeded into 6-well plates and transfected the next day. 60 h later, cells were harvested for immunoblotting.

### Immunoblotting

Cells were lysed in 1 × SDS loading buffer containing 50 mM Tris pH 6.8, 2% SDS, 0.025% bromophenol blue, 10% glycerol, and 5% BME. The concentration of total proteins was assayed by BCA method. Protein samples were separated by SDS-polyacrylamide gel electrophoresis (SDS–PAGE) and then transferred onto polyvinylidene fluoride (PVDF) membranes, blocked with 5% non-fat milk in TBST for 1 h at room temperature. The membranes were washed with TBST three times for 5 min and then incubated with primary antibodies (4°C overnight) and HRP-conjugated secondary antibodies. ECL solution and image acquisition equipment (5200S Imager) were from Tanon Science & Technology Co., Ltd. The following primary antibodies were used: anti-LATS2 (CST, 1:1000, 5888S), anti-YAP (CST, 1:1000, 14074S), anti-pYAP (S127) (CST, 1:1000, 4911S), anti-CYR61 (Santa, 1:1000, sc-13011), and anti-vinculin (CST, 1:1000, 13901s).

### Immunohistochemistry

Paraffin embedded tissue specimens were sectioned, dewaxed, and rehydrated. Antigen retrieval was performed in 10 mM sodium citrate buffer (pH 6.0) at 95–100°C for 20 min. Endogenous peroxidase activity was blocked by 3% H_2_O_2_ for 30 min. Sections were then blocked in 5% BSA for 1 h and incubated with primary antibodies overnight. After extensive washing, the sections were incubated with secondary antibodies at room temperature for 1 h. DAB solution was applied and hematoxylin was used for counterstaining. Anti-YAP (CST, 1:200) was used as a primary antibody. Staining results were visualized with Zeiss Axiocam 208 color. Quantification was conducted to measure the protein expression.

### DNA Isolation and Bisulfite Conversion

Genomic DNA of glioma was isolated from frozen LGG tissues using DNA/RNA/protein Extraction Kit (DP423) (Tiangen, Beijing, China) according to the manufacturer’s protocol. EZ DNA Methylation-Startup Kit (Zymo Research) was utilized to perform sodium bisulfite modification of DNA following the manufacturer’s instructions. This converts cytosine residues to uracil in single-stranded DNA while leaving methylated cytosine unchanged.

### Methylation-Specific PCR

The methylation status of *LATS2* was tested by Methylation-specific PCR utilizing both methylated and unmethylated specific sets of primers: 5′-GTT GGA GTT GTT GTT GGT TTC-3′ (forward) and 5′-CGA ATA TCC CAC TTA AAT CTA CG-3′(reverse) for methylated reaction (PCR products, 131 bp) and 5′-GTT GGA GTT GTT GTT GGT TTT G-3′ (forward) and 5′-AAA TAT CCC ACT TAA ATC TAC ACT-3′ (reverse) for unmethylated reaction (PCR product, 130 bp). PCR amplification was carried out on T-100 Thermal Cycler (Bio-Rad) using Taq DNA polymerase (Vazyme Biotech Co., Ltd., China) in a total volume of 10 μL. 5% DMSO was added to enhance the specificity and yield of PCR reactions. DNA samples were initial denatured at 95°C for 5 min, and was followed by 36 cycles of denaturing at 95°C for 30 s, annealing at 54°C (for methylated reaction) or 59°C (for unmethylated reaction) for 30 s, and extension at 72°C for 45 s. A final extension step at 72°C for 5 min was added for all reactions. Both positive and negative controls were included. Polymerase chain reaction products were subsequently electrophoresed on 2% agarose gels and visualized with image equipment from Tanon Science & Technology Co., Ltd.

## Data Availability Statement

Publicly available datasets were analyzed in this study. This data can be found here: https://portal.gdc.cancer.gov.

## Ethics Statement

The studies involving human participants were reviewed and approved by Ethics Committee of the Huashan Hospital of Fudan University. The patients/participants provided their written informed consent to participate in this study.

## Author Contributions

YG and F-XY designed the study and wrote the manuscript. YG, YuW, YeW, JL, XW, MM, WH, and YL performed experiments and data analysis. All authors contributed to the article and approved the submitted version.

## Conflict of Interest

The authors declare that the research was conducted in the absence of any commercial or financial relationships that could be construed as a potential conflict of interest.
