# Supplementary material for: Hypermethylation of LATS2 Promoter and Its Prognostic Value in IDH-Mutated Low-Grade Gliomas
Source: Front Cell Dev Biol. 2020 Oct 22;8:586581. doi: 10.3389/fcell.2020.586581 (PMC7642219; doi:10.3389/fcell.2020.586581)
Supplement: Supplementary file 1 [file Data_Sheet_1.docx]

Supplementary Material

# Supplementary Figures and Tables

## Supplementary Figures

**
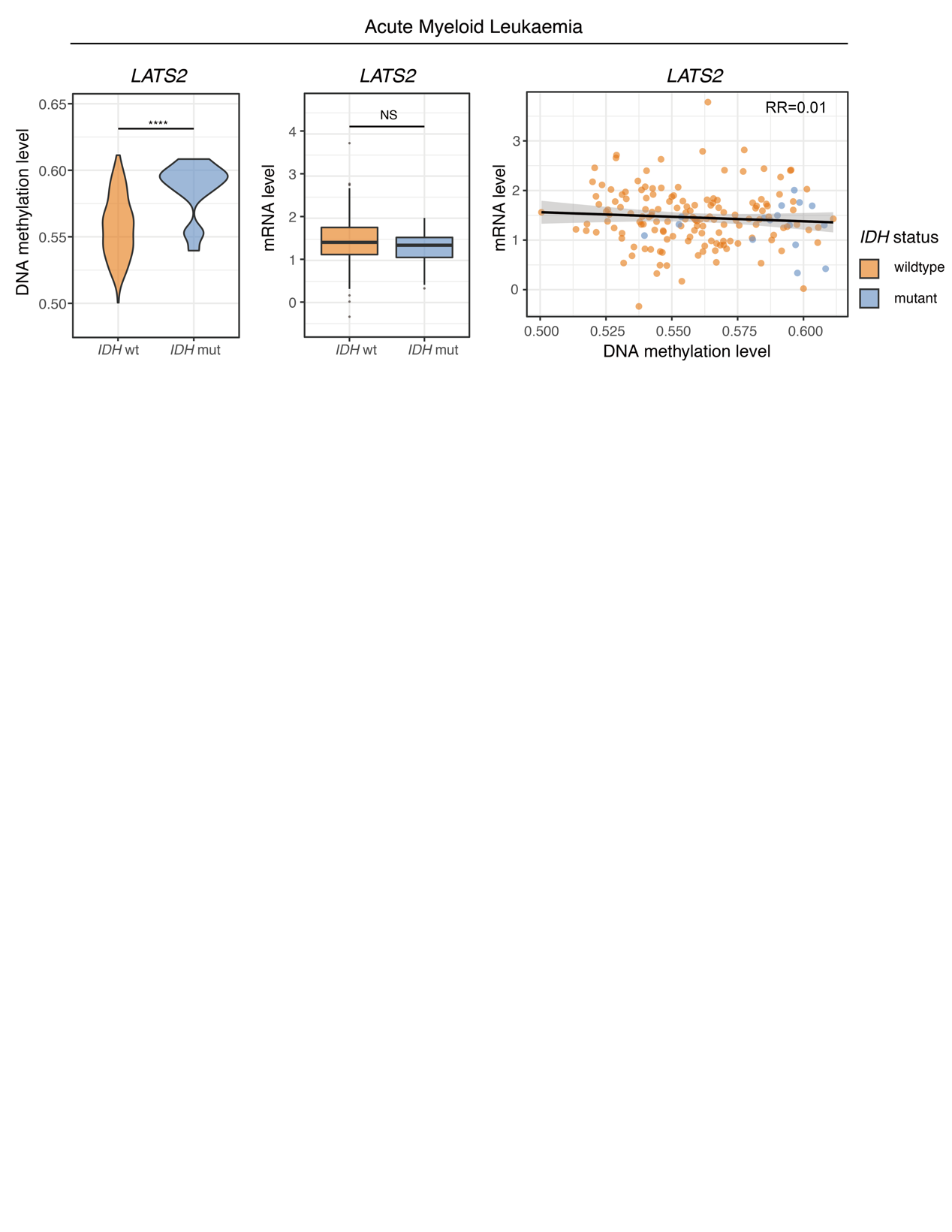
 Supplementary Fig. 1** *LATS2* status in AML. *LATS2* methylation level is increased in *IDH*-mutant LGG (left), while *LATS2* mRNA level is not changed in *IDH*-mutant LGG (middle). No correlation between *YAP* mRNA level and *LATS2* methylation level was observed(right). RR indicates R squared value of linear regression. Mean and standard error were presented (****<0.00005, ns: not significant, t test).


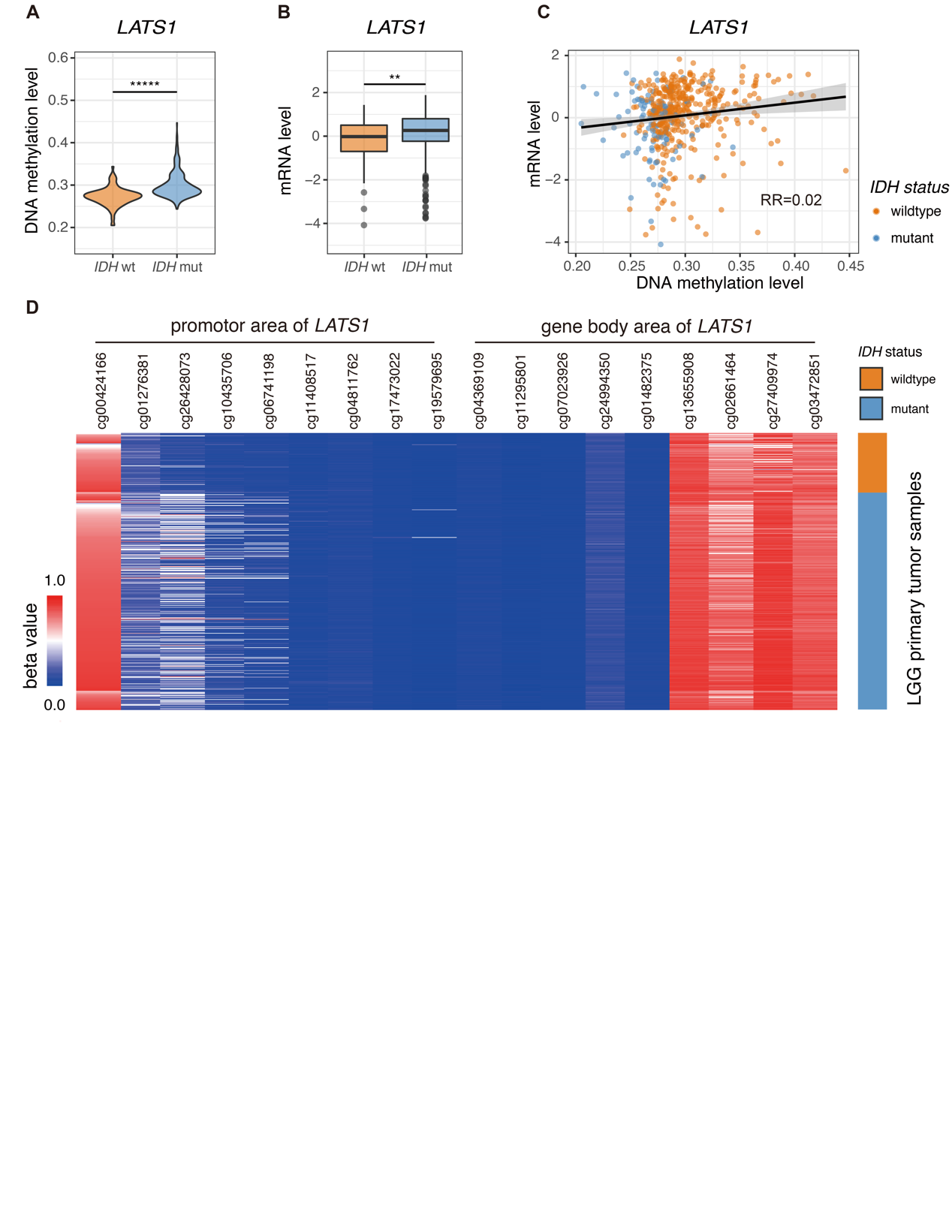


**Supplementary Fig. 2** Promoter methylation and expression level of *LATS1* in IDH-mutant LGG. (A) *LATS1* methylation level is increased in IDH-mutant LGG. (B) *LATS1* mRNA level is increased in IDH-mutant LGG. (C) Correlation between *LATS1* methylation level and *LATS1* mRNA level. RR indicates R squared value of linear regression. (D) Methylation level of different CpG islands in *LATS1* promoter and gene body area. Mean and standard error were presented (**p < 0.005, *****p < 0.000005, t test).

**
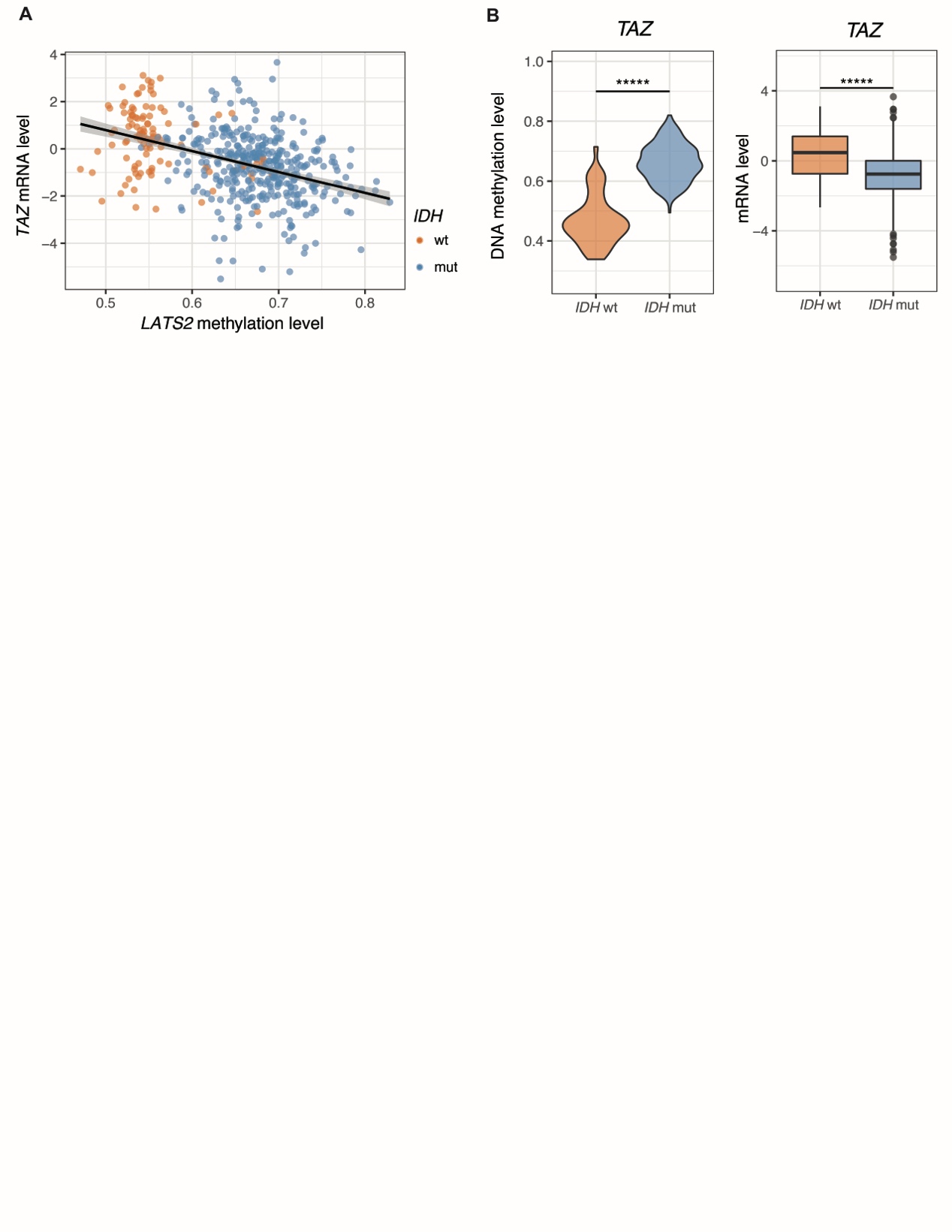
 Supplementary Fig. 3** *TAZ* is downregulated in *IDH*-mutant LGG. (A) Correlation between *TAZ* mRNA level and *LATS2* methylation level. (B) *TAZ* methylation level is increased in *IDH*-mutant LGG (left). *TAZ* mRNA level is decreased in *IDH*-mutant LGG (right). Mean and standard error were presented (****<0.00005, t test).

**
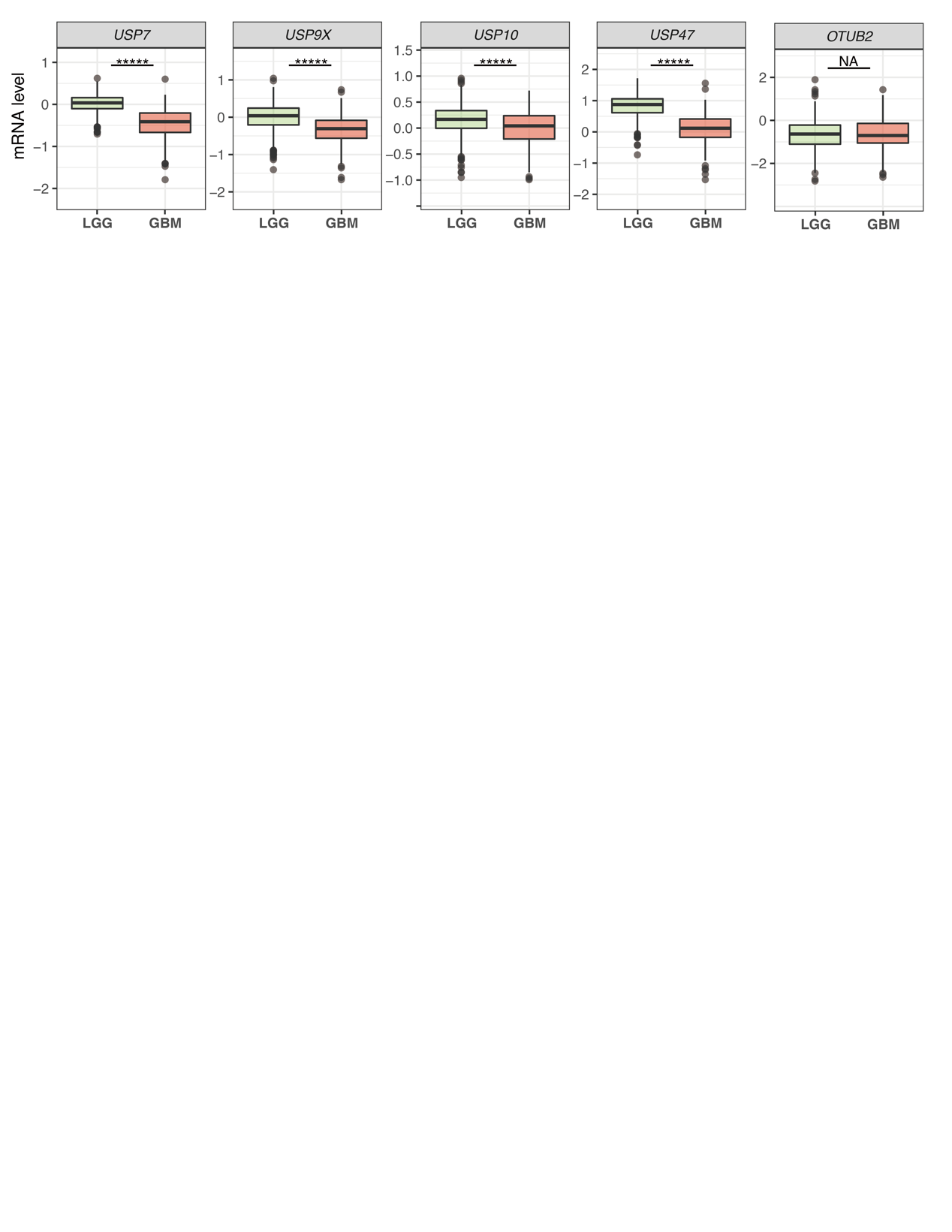
 Supplementary Fig. 4** YAP deubiquitinases are upregulated in *IDH*-mutant LGG. Mean and standard error were presented (*****<0.000005, ns: not significant, t test).

**
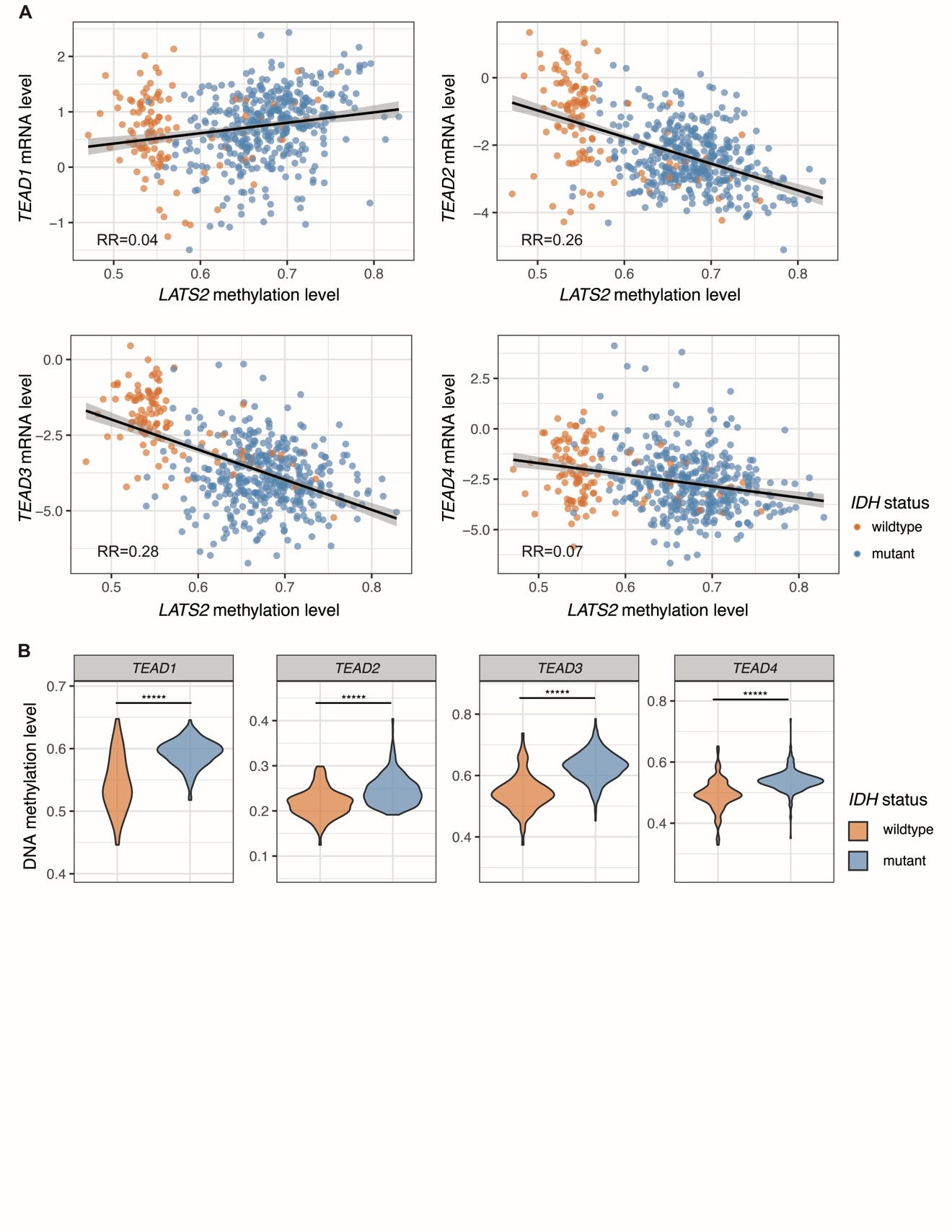
**

**Supplementary Fig. 5** (A) Correlation between *TEAD1-4* mRNA level and *LATS2* methylation level. (B) The methylation of *TEADs* are increased in *IDH*-mutant LGG, *****p <0.000005, t test

**
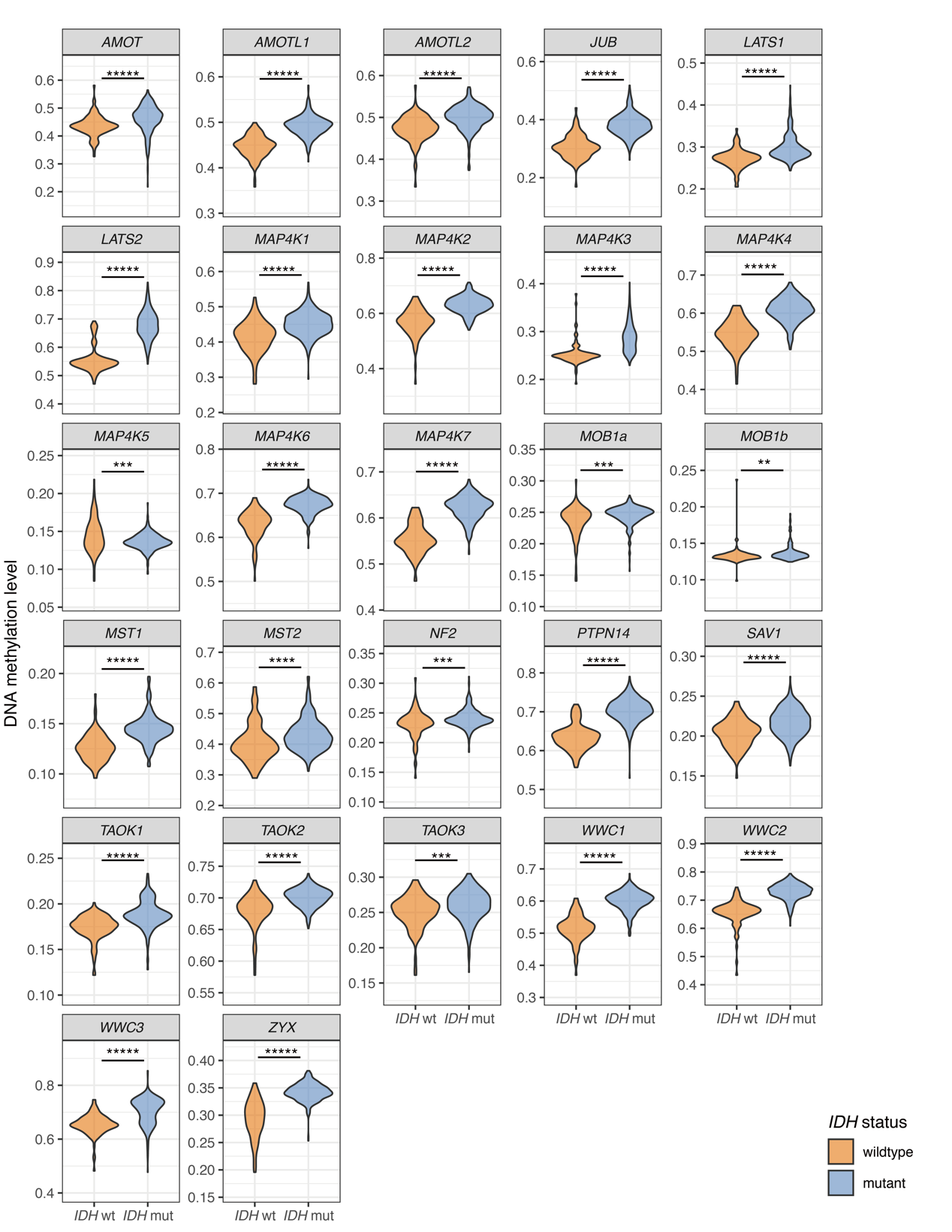
**

**Supplementary Fig. 6** Methylation level of Hippo pathway regulators in *IDH-*wildtype and mutant LGG, **p <0.005, ***p <0.00005, *****p <0.000005. t test.

**
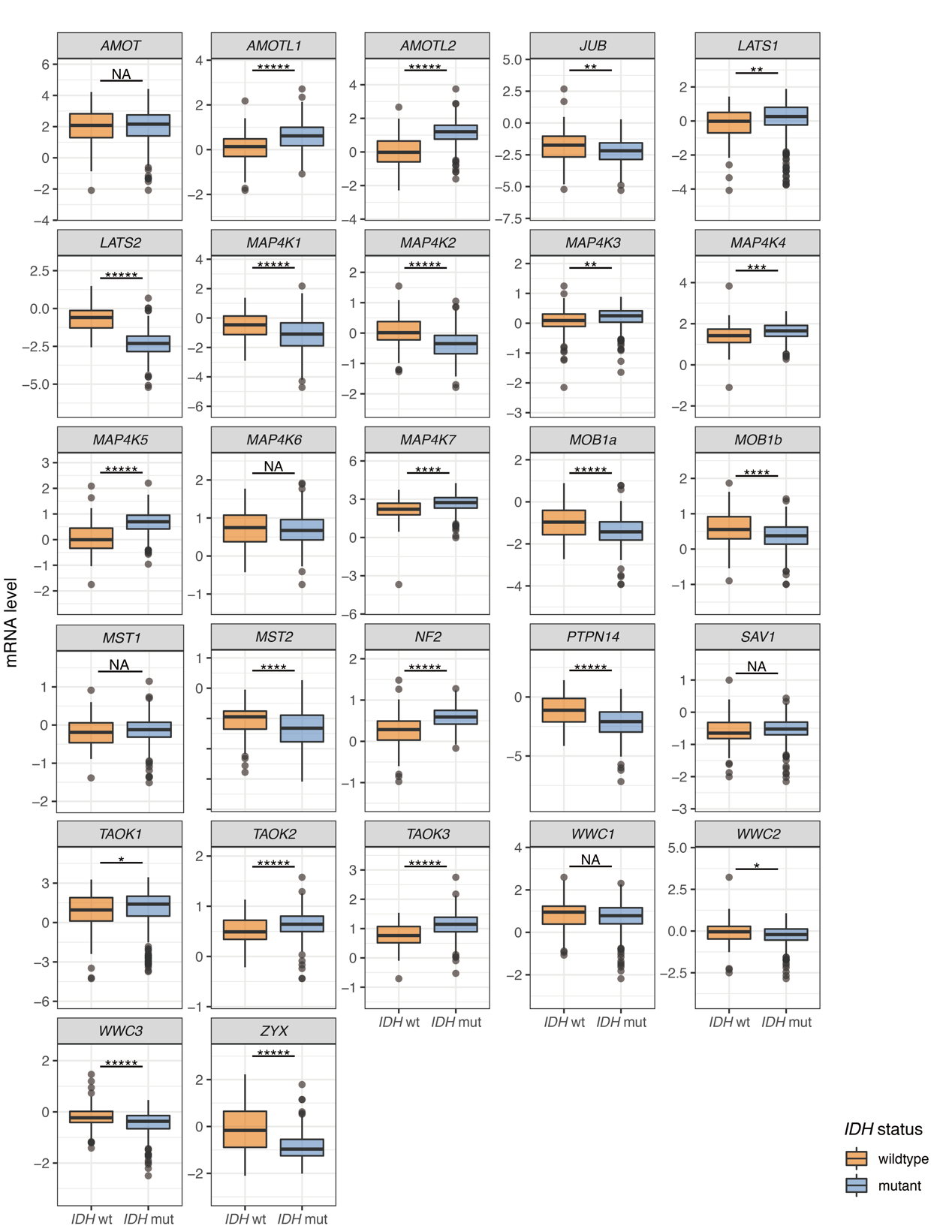
**

**Supplementary Fig. 7** mRNA level of Hippo pathway regulators in *IDH-*wildtype and mutant LGG. Mean and standard error were presented (*p<0.05, **p<0.005, ***p<0.0005, ****<0.00005, *****p <0.000005, ns: not significant, t test).

**
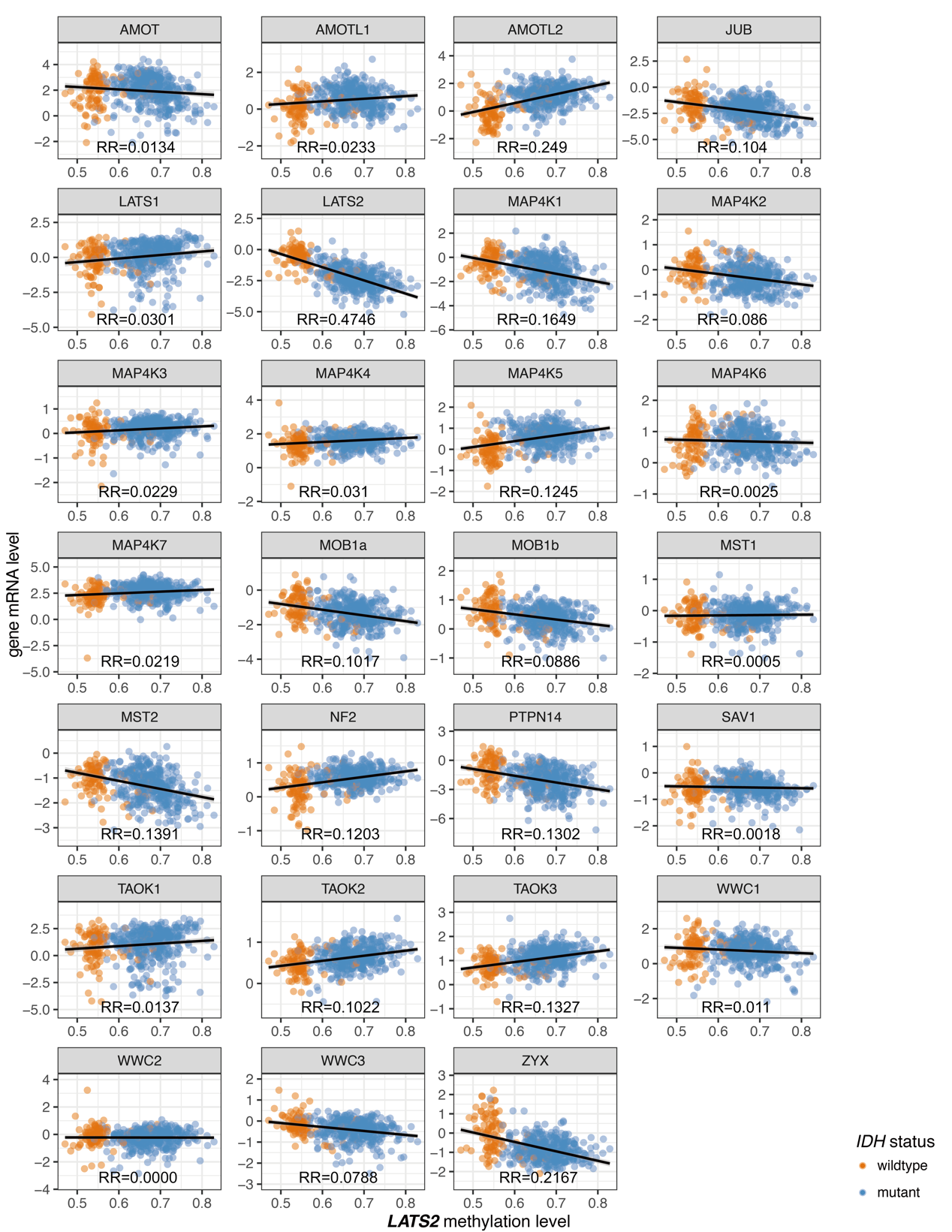
**

**Supplementary Fig. 8** Correlation between gene mRNA level and *LATS2* methylation level of Hippo pathway regulators. RR indicates R squared value of linear regression.

**
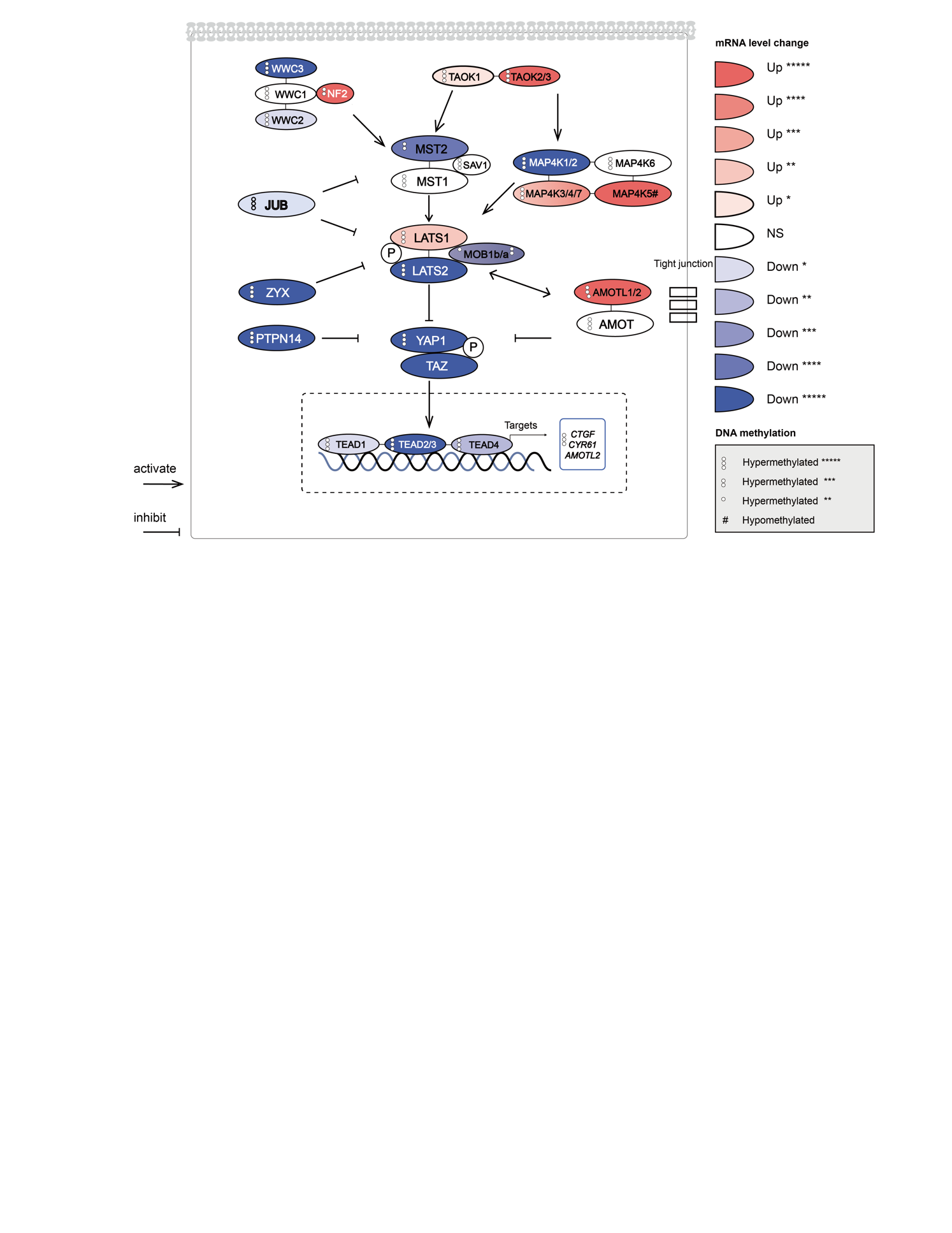
**

**Supplementary Fig. 9** Methylation and transcriptional status of Hippo pathway.

## Supplementary Tables

**Supplementary Table 1.** Hippo target gene list used in Figure 2B and Figure 3A

| AMOTL2 | ANKRD1 | AXL | BICC1 | BIRC5 | CDC20 |
| --- | --- | --- | --- | --- | --- |
| CDKN2C | CENPF | COL4A3 | CRIM1 | CTGF | CYR61 |
| DAB2 | DDAH1 | ASAP1 | DLC1 | DUSP1 | DUT |
| ECT2 | EMP2 | ETV5 | FGF2 | FLNA | FSCN1 |
| FSTL1 | GADD45B | GAS6 | GGH | GLS | HEXB |
| HMMR | AGFG2 | ITGB2 | ITGB5 | LHFP | MARCKS |
| MDFIC | NDRG1 | PDLIM2 | PHGDH | PMP22 | SCHIP1 |
| SDPR | SERPINE1 | SGK1 | SH2D4A | SHCBP1 | SLIT2 |
| STMN1 | TGFB2 | TGM2 | THBS1 | TK1 | TNNT2 |
| TNS1 | TOP2A | TSPAN3 |  |  |  |

**Supplementary Table 2.** Clinical features of IDH-wildtype and mutant LGG patients

| Characteristics | total | Low *LATS2* methylation | High *LATS2* methylation | P value |
| --- | --- | --- | --- | --- |
| Overall | 503 | 251 | 252 |  |
| Age |  |  |  | 0.0501 |
| <40 | 219 | 119 | 100 |  |
| 40-60 | 213 | 93 | 120 |  |
| >60 | 70 | 39 | 31 |  |
| Gender |  |  |  |  |
| female | 224 | 116 | 108 | 0.5042 |
| Male | 279 | 135 | 144 |  |
| Histological type |  |  |  |  |
| Astrocytoma | 190 | 118 | 72 | 0.0000 |
| Oligoastrocytoma | 128 | 65 | 63 |  |
| Oligodendroglioma | 185 | 68 | 117 |  |
| Histologic grade |  |  |  |  |
| G2 | 241 | 105 | 136 | 0.0131 |
| G3 | 261 | 146 | 115 |  |
| Tumor location |  |  |  |  |
| Posterior Fossa, Brain Stem | 1 | 1 | 0 | 0.0042 |
| Posterior Fossa, Cerebellum | 2 | 2 | 0 |  |
| Supratentorial, Frontal Lobe | 296 | 125 | 171 |  |
| Supratentorial, NOS* | 8 | 5 | 3 |  |
| Supratentorial, Occipital Lobe | 8 | 4 | 4 |  |
| Supratentorial, Parietal Lobe | 45 | 28 | 17 |  |
| Supratentorial, Temporal Lobe | 142 | 86 | 56 |  |
| *IDH* status |  |  |  |  |
| mutant | 395 | 150 | 245 | 0.0000 |
| wildtype | 108 | 101 | 7 |  |

*NOS, Not Otherwise Specified
